# Reducing Waste: Instrument Recycling in the Emergency Department

**DOI:** 10.5811/westjem.52924

**Published:** 2026-05-13

**Authors:** Esther H. Chen, Kristie Taguma, Newton Addo, John K. Quinn

**Affiliations:** * University of California, San Francisco, Department of Emergency Medicine, San Francisco, California; † Zuckerberg San Francisco General Hospital, San Francisco, California; ‡ Kaiser Foundation Hospital-San Leandro, Department of Emergency Medicine, San Francisco, California; § University of Washington, Department of Emergency Medicine, Seattle, Washington

## Abstract

**Introduction:**

Emergency medicine (EM) residency programs are required to teach quality improvement (QI), yet few adopt a sustainability lens to QI despite broad recognition of the importance of climate sustainability in healthcare. To address this gap, some programs have piloted innovative approaches such as sustainability QI electives or projects, although evidence of their effectiveness remains limited.

**Methods:**

We designed and implemented a sustainability QI initiative to recycle used instruments from three bedside procedure kits (laceration repair, incision and drainage, chest tube placement) commonly used in the emergency department to reduce waste. Our goal was to describe the effectiveness of a financial intervention on instrument recycling by comparing the differences in recycling rates between the baseline and incentive periods using a quasi-Poisson regression analysis.

**Results:**

At the end of the first year of instrument recycling, the recycling rate was 9%. Providing a financial incentive to residents over a two-year period significantly increased the recycling rate to a mean of 24% (standard deviation 13), with a rate ratio of 3.03 (95% CI 1.57–5.85), *P* < .001. While the residents did not meet their recycling target of 50% to receive the incentive payment, their overall recycling rate increased.

**Conclusion:**

Providing a financial incentive to residents for recycling efforts was modestly successful in encouraging residents to participate in an instrument recycling initiative. Motivating busy clinicians to engage in sustainable practice is challenging; projects that prioritize systems-level changes may be more effective than those that require changes in individual clinical practices.

## INTRODUCTION

Emergency medicine (EM) residency programs are required to teach quality improvement (QI) concepts, preparing residents to identify opportunities to enhance clinical practice through streamlined processes and improved patient safety.^1^,^2^ However, few programs approach QI using the lens of climate sustainability or incorporate sustainable QI and climate change and health (CCH) topics into their core curricula.^3^ One program implemented a climate health elective rotation in which EM residents learn sustainable clinical practice principles and apply those principles to design and implement a sustainability QI capstone project.^4^ While promising, curricular interventions have been found to be least effective in promoting sustainable behavior change, whereas social comparisons or financial incentives were the most effective.^5^

Recognizing a curricular gap in our own program, we implemented an instrument recycling sustainability QI initiative to teach residents about reducing waste generated during routine clinical practice. Emergency departments (ED) contribute to greenhouse gas emissions by generating a significant amount of waste, deviating from hospital waste disposal policies (ie, throwing waste in regulated waste red bags), or mixing waste with recyclable paper or plastics.^6^ Waste reduction strategies such as increased recycling and reprocessing of single-use devices may reduce carbon dioxide emissions and lead to financial cost-savings.^7^

Clinician-level strategies to reduce waste-related emissions include recycling of single-use materials and reducing the disposal of unused equipment.^8^ Our ED had already been collecting unused supplies from procedure kits for resident teaching and international distribution.^9^ To further reduce clinician-generated waste, we decided to implement recycling of instruments from procedure kits used during clinical care. We describe our experience with providing a financial incentive to increase instrument recycling in a busy ED setting, aiming to teach residents to approach sustainability QI with the same rigor as patient-focused QI projects.

## METHODS

### Setting and Participants

The EM residency program has 60 postgraduate year 1–4 residents who spend about 50% of their clinical time at the county hospital and trauma center ED (annual census about 65,000 patients). This project was approved as exempt from institutional review.

### Design

In June 2022, the county hospital implemented recyclable instruments in the commonly used bedside procedure kits (ie, laceration repair, incision and drainage, chest tube placement). Rather than disposing instruments into the room’s sharps container, instruments could be deposited into a recycling bin in the soiled utility room located in each clinical area. Instruments were collected and recycled by the instrument manufacturer when the bins were completely full. The hospital incurred no additional cost for instrument recycling. This recycling initiative was announced in July 2022 to faculty at faculty meeting and to residents during residency conference.

In July 2023, we proposed that the instrument recycling project be part of the hospital’s pay-for-performance (P4P) program for QI and sustainability QI initiatives. This pay-for-performance program has been successful in engaging trainees to participate in QI efforts for a financial incentive.^10^ The instrument recycling project was approved for the 2023–2025 academic years, in which all EM residents would receive $400 at the end of the year for meeting their monthly instrument recycling goal for at least six months of the academic year.

### Outcome Measurements and Analysis

Our primary outcome was the monthly recycling rate. To establish a baseline, we measured the weight of recycled instruments in July 2023 using the ED standing scale and continued monthly measurements during the first week of each month. We calculated the per-unit weight by weighing the recyclable instruments of each kit. Every month, we calculated the expected weight of recycled instruments by multiplying the per-unit weight by the number of kits replaced from the hospital’s central supply (assuming 100% of the instruments were recycled). We defined the recycling rate as the actual weight of recycled instruments divided by the expected weight of the instruments from the replaced kits. Additionally, from July 2024–June 2025, we recorded the monthly number of laceration repair, incision and drainage, and chest tube placement procedure notes documented in the electronic health record to provide another estimate of the total weight of used kits.

**Population Health Research Capsule**

What do we already know about this issue?

*Financial incentives may be more effective than curricular interventions to promote sustainable, clinician-level behavior change*.

What was the research question?

*We tested the effectiveness of offering a financial incentive to residents to increase instrument recycling*.

What was the major finding of the study?

*Instrument recycling increased from 9% to 24%, with a rate ratio of 3.03 (95% CI 1.57–5.85), P < .001*.

How does this improve population health?

*Financial incentives could promote sustainable clinical practice and reduce waste-related emissions*.

Data are presented using descriptive statistics with means and standard deviations as appropriate. We used a generalized linear model with a quasi-Poisson distribution, month of observation, and a log-link function to assess the differences in recycling rates between the baseline and incentive periods. We report rate ratios (RR) and 95% confidence intervals, with the baseline period serving as the reference group. All analyses were performed with R v4.4 (The R Foundation for Statistical Computing, Vienna, Austria).

## RESULTS

At the end of the first year of instrument recycling (July 2022–July 2023), the recycling rate was 9%. Providing a financial incentive to residents from July 2023–June 2025 significantly increased the recycling rate to a mean of 24% (SD 13), with a RR of 3.03 (95% CI 1.57–5.85), *P* < .001. The monthly instrument recycling rate (Figure) and weight of recycled instruments were variable (Table). The residents did not meet their recycling target of 50% of instruments recycled over the two-year period and, therefore, did not receive an annual $400 incentive payment.

## DISCUSSION

Our sustainability QI project was successful in increasing the overall instrument recycling rate in the ED although there was month-to-month variability. Despite not reaching the proposed recycling target of 50%, the overall instrument recycling rate increased from 9% to 24%. and a sustained improvement was observed in the project’s second year. We concurrently implemented a climate change and health curriculum into weekly conferences, using these sessions to provide quarterly reminders to residents about the instrument recycling program as a way to reduce environmental waste. In addition, resident QI leaders highlighted the program with reminders during shift handoffs and provided orientation on the recycling workflow to non-EM rotators and medical students.

However, this project also highlighted the challenge of implementing a sustainability QI project that depended on changing individual behavior in a busy clinical environment and the cognitive load of adopting a new process. The recycling workflow required already busy residents to collect and transport their instruments to the soiled utility room rather than simply depositing them directly into the sharps containers located in each patient room. Residents rotate to different clinical sites every 2–4 weeks, which can potentially lead to confusion regarding which QI and sustainability QI initiatives were implemented at each location. Faculty may also be unable to consistently remind residents about instrument recycling when supervising procedures, particularly if they are not present during cleanup. Additionally, more than 50% of resident shifts are staffed by non-EM residents, who may be unaware of recycling efforts. Finally, residents face competing priorities, such as optimizing patient care, which may overshadow any consideration of sustainable clinical practices.

While not meeting the recycling goal was disappointing, this project showed that sustainability QI initiatives targeting system-level change rather than focusing on changing practice at an individual level may be more effective in achieving desired outcomes. For example, a prior ED sustainability QI initiative implemented automatic double-sided printing of after-visit summaries by adjusting the default settings of all departmental printers. This system-wide change eliminated the need to modify settings manually and effectively reduced paper use by preventing single-sided printing. For the instrument recycling initiative, even though financial incentives improved the recycling rate, we might have seen greater success if we had been able to simplify the instrument disposal workflow by installing recycling bins in each patient room. Alternatively, EDs could take steps to more fully integrate climate sustainability into their culture and expand their clinicians’ knowledge of sustainable practices with consistent communication about sustainability QI initiatives, in the same way as patient-focused QI initiatives.

## LIMITATIONS

This project had several limitations. It was conducted with a single residency program at an institution that benefits from a pay-to-perform improvement program and dedicated QI faculty mentorship, which may limit generalizability to other settings. In addition, we encountered specific hospital system challenges during our study period, which limited our interventions (ie, installing recycling bins in each patient room). Furthermore, we implemented a concurrent quarterly climate change and health lecture series during residency conference to teach residents to manage climate-related health emergencies and to remind residents about the instrument recycling initiative. These reminders may also have increased the recycling efforts.

## CONCLUSION

Providing a financial incentive to residents for recycling efforts was modestly successful in encouraging them to participate in an instrument-recycling sustainability quality improvement initiative. Motivating busy clinicians to engage in sustainable practice is challenging; projects that prioritize systems-level changes may be more effective than those that require changes in individual clinical practices.

## Figures and Tables

**Figure:**
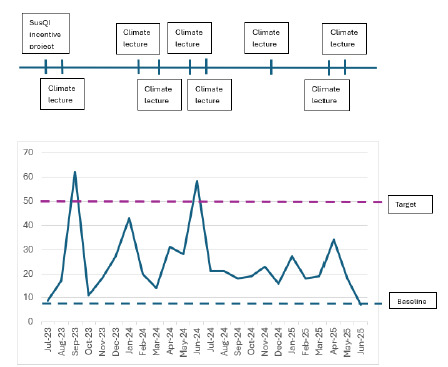
Monthly instrument recycling rate with the timeline of the climate lecture series. *SusQI*, sustainability quality improvement.

**Table:** Monthly recycling rate and weight of recycled instruments in the emergency department.

Date	Recycling rate (%)	Weight (kg)
Jul 2023	9	21 (total weight from 7/22–7/23)
Aug 2023	17	2
Sep 2023	62	2.5
Oct 2023	11	1.5
Nov 2023	18	1.9
Dec 2023	27	2.9
Jan 2024	43	5.9
Feb 2024	20	2.6
Mar 2024	14	1.7
Apr 2024	31	3.7
May 2024	28	3.3
Jun 2024	58	8
Jul 2024	21	3.9
Aug 2024	21	2.5
Sep 2024	18	2.7
Oct 2024	19	3.1
Nov 2024	23	3.9
Dec 2024	16	2.5
Jan 2025	27	4.4
Feb 2025	18	3.1
Mar 2025	19	3
Apr 2025	34	4
May 2025	18	3
Jun 2025	7	3.1

*kg*, kilogram.
